# Genotoxic Effects of Lead and Their Impact on the Expression of DNA Repair Genes

**DOI:** 10.3390/ijerph19074307

**Published:** 2022-04-03

**Authors:** Sirirak Hemmaphan, Narisa K. Bordeerat

**Affiliations:** 1 Graduate Program in Biomedical Sciences, Faculty of Allied Health Sciences, Thammasat University, Rangsit 12121, Thailand; sirirak.h@allied.tu.ac.th; 2 Department of Medical Technology, Faculty of Allied Health Sciences, Thammasat University, Rangsit 12121, Thailand

**Keywords:** lead, genotoxicity, gene expression, DNA repair

## Abstract

Exposure to lead (Pb) continues to be a significant worldwide problem. Pb is a highly poisonous heavy metal affecting several organ systems in the body. Although Pb has been shown to be genotoxic to experimental animals and humans, the underlying mechanisms are still not understood. An indirect mechanism related to the inhibition of DNA repair systems by Pb has been suggested. Heavy metals can interfere with the activities of several proteins and gene expressions. Recent studies gathered in this review article demonstrated an altered expression of DNA repair genes due to Pb toxicity. However, their findings are conflicting. Furthermore, the interaction of Pb and epigenetic mechanisms regulating gene expression may have a crucial role in the inhibition of DNA repair systems. Therefore, additional studies are needed to evaluate these findings and to obtain a complete picture of the genotoxic properties of Pb and the underlying mechanisms that may have a crucial role in carcinogenesis.

## 1. Introduction

Lead (Pb) is one of the most widely used heavy metals in several industries for the manufacturing process of Pb-based products due to its physical and chemical properties, such as high density, softness, malleability, and poor conductibility. Thus, Pb can be found in workplaces and other contaminated environments. Pb toxicity can occur via both nonoccupational and occupational exposure through inhalation, ingestion, and dermal absorption. Absorbed Pb enters the plasma and then moves rapidly to various body areas Pb is exchanged primarily among three areas, including the blood, mineralizing tissues (teeth and bones), and soft tissues (liver, kidneys, lungs, brain, spleen, muscles, and heart), contributing to Pb accumulation and the induction of its mechanisms of action in several organ systems, such as the nervous, hematological, digestive, cardiovascular, skeletal, reproductive, and excretory systems [1]. Thus, Pb is a highly poisonous heavy metal affecting almost every organ in the body. Moreover, the genotoxic effects of lead have been studied for a long time. The genotoxic endpoints induced by Pb have been well demonstrated in different test systems. Pb was found to produce positive responses in several biological and biochemical tests for DNA breaks and lesions, mutation, and DNA oxidative damage [2,3,4,5]. However, the exact mechanisms are still largely unknown. Previous studies suggested that the mechanics of its genotoxicity could be involved with indirect mechanisms, such as the inhibition of DNA repair systems [2,3,4,5,6].

DNA molecules are continuously damaged by both endogenous and exogenous genotoxic factors. These genotoxic factors contribute to DNA damage and genome instability, affecting transcription and replication, and they can be inherited by daughter cells. However, cells have a repair process known as the DNA damage response (DDR) for the recognition and repair of this DNA damage. The DNA repair mechanisms include base excision repair (BER), nucleotide excision repair (NER), mismatch repair (MMR), homologous recombination (HR), non-homologous end joining (NHEJ), translesion synthesis, and DNA interstrand crosslink repair [1]. In DNA repair systems, several DNA repair genes and their encoded proteins are responsible for monitoring chromosomes by correcting the damaged nucleotide residues in specific repair pathways, for example 8-oxoguanine DNA glycosylase 1 (hOGG1), X-ray repair cross-complementing protein 1 (XRCC1), and Excision Repair 1(ERCC1), which play a crucial role in ROS-induced DNA repair pathways. Importantly, the inactivation of DNA repair genes contributes to a deficiency in DNA repair and the accumulation of DNA damage that promotes tumorigenesis [7]. In addition, epigenetics involves gene expression and regulation without DNA sequence changes. Transcriptional regulation is administered through important epigenetic pathways, dictated primarily by DNA methylation, RNA regulation, and the posttranslational modification (PTM) of histones [8]. Several previous studies demonstrated an interaction between heavy metals and the aberrant expression of DNA repair genes via epigenetic mechanisms, such as aberrant DNA methylation, modified histone modification, and the altered expression profiles of microRNAs (miRNAs) and long non-coding RNAs (lncRNAs) [9,10,11]. Moreover, some studies have shown that heavy metal-impaired DNA repair was mediated by aberrant expression through mutation in the exon of DNA repair genes.

Heavy metals can interfere with the activities of several proteins and alters the expression pattern of numerous genes [12,13]. Recent studies also reported a role of Pb toxicity in the impairment of DNA repair systems. This review gathered evidence of the impact of Pb toxicity on the altered expression of DNA repair genes. Although the results are conflicting, these findings reinforce the need for the investigation of the mechanism of genotoxic effects of Pb related to the inhibition of DNA repair systems that promote cancer development.

## 2. Genotoxicity of Lead

The International Agency for Research on Cancer (IARC) has classified Pb as a possible human carcinogen (group 2B) and its inorganic compounds as probable human carcinogens (group 2A). The genotoxic effects of Pb have been investigated for many years and include chromosome aberration (CA), mutation, DNA breakage, and DNA synthesis inhibition [14,15]. According to previous studies, the end-points of the genotoxic effects of Pb have been well-demonstrated in different in vitro, in vivo, and epidemiological studies. Pb has been tested and found to exhibit positive responses in biological and biochemical tests for DNA lesions, such as structural and numerical CA, sister chromatid exchanges (SCE), micronucleus (MN) tests, and DNA strand breaks using the single-cell gel electrophoresis (comet) assay [2]. Moreover, hypoxanthine-guanine phosphoribosyl-transferase (HPRT) gene and T-cell receptor (TCR) mutation assays, most frequently used to determine the mutations caused by mutagenic agents in somatic cells, also indicated the genotoxic effect of Pb [2].

The genotoxic endpoints induced by Pb have been well-known for a long time. However, the genotoxic properties and mechanisms underlying the genotoxic effects of Pb are still unclear. It has been suggested that the mechanisms of the genotoxic effects of Pb could be involved with indirect mechanisms, such as the induction of oxidative stress contributing to DNA damage, the inhibition of DNA repair, the formation of DNA and/or protein crosslinks, and the regulation of tumor suppressor and promoter genes [6,16,17,18,19].

The major mechanism of Pb toxicity is primarily involved in oxidative stress, described as an imbalance between the generation of reactive oxygen species (ROS) and the ability of antioxidants [20]. Pb is capable of inhibiting the activities of antioxidant enzymes by interacting with a functional sulfhydryl (SH) group in antioxidant enzymes, such as δ-aminolaevulinic acid dehydrase (δ-ALAD), superoxide dismutase (SOD), catalase (CAT), glutathione peroxidase (GPx), and glucose-6-phosphate dehydrogenase (G6PD) [21,22,23]. Inhibition of δ-ALAD, which catalyzes the condensation of delta-aminolaevulinic acid (δ-ALA) to porphobilinogen (PBG) in the pathway for heme synthesis, leads to accumulation of δ-ALA [24]. This eventually stimulates ROS production and the generation of 4,5-dioxovaleric acid, which is an efficient alkylating agent of the guanine moieties within both nucleoside and isolated DNA [25]. As a consequence of alkylation, single-strand breaks and quinine oxidation were produced with an increase in the level of 8-hydroxy-2′-deoxyguanosine (8-OHdG) or 8-oxo-7,8-dihydro-2′-deoxyguanosine (8-oxodG) [25]. 8-OHdG is one of the predominant forms of oxidative lesions and has been widely used as a biomarker for oxidative stress [26]. Several studies demonstrated a positive relation between 8-OHdG and Pb [15,27,28]. The DNA repair machinery plays the crucial role of protecting the cells from DNA damage generated by exposure to carcinogens and cytotoxic agents, as well as heavy metals. A previous study suggested that Pb substitutes calcium and zinc in enzymes involved in DNA processing and repair, resulting in an enhancement in genotoxicity when combined with other DNA-damaging agents such as tobacco smoke or UVA [2]. Interestingly, an abnormal DNA repair capacity was reported in lead-exposed workers [29,30].

## 3. Study on the Effect of Lead on DNA Repair-Related Genes

### 3.1. In Vitro Studies

Most of the studies determined the effects of Pb on DNA repair gene expression with different cell systems, methodologies, and results, as shown in Table 1.

Gadhia et al. (2012) evaluated changes in expressions of genes responsible for DNA repair in Pb-exposed mouse embryonic stem (mES) cells [9]. Their results showed that cells exhibited significant decreases in mRNA expressions of OGG1, Top3a, and Rad18 after exposure to lead acetate (PbAc) at IC50 concentration for 1 h. PbAc-exposed lymphoblastoid TK6 cells showed obvious decreases in DNA repair protein levels, including XRCC1 at 12 h; hOGG1 at 6, 12, and 24 h; BRCA1 at 12 and 24 h; and XPD at 6 h of exposure [31]. Moreover, a previous in vitro study using plant cells also demonstrated a change in DNA repair gene expression after Pb treatment. The mRNA expression of POLD1 was significantly decreased after 5 µM and 15 µM PbAc treatment for 12 h in root-tip cells of *Allium cepa* var. *agrogarum* L. [32]. In contrast, no significant change in the DNA repair gene expression was documented. Abdullah et al. (2014) reported the unchanged expressions of ERCC3, XRCC14, and RAD 51 in stem cells isolated from deciduous teeth (SCDs), permanent teeth (DPSCs), periodontal ligaments (PDLs), and bone marrow (BM-MSCs) after 24 h of exposure to lead nitrate (Pb [NO_3_]_2_) at a concentration of 160 µM [33]. Furthermore, Wang et al. (2013) reported a significant induction of DNA repair protein APE1 expression was observed in CL3 human lung cancer cells following exposure to 10–100 µM PbAc for 30 min and 24 h [34].

**Table 1:** In vitro studies on the effects of Pb on the expression of DNA repair-related genes.

Test System	Substance	Treatment	DNA Repair Gene	Method	Result	Reference
Mouse embryonic stem (mES) cells	Lead acetate	0.02 mg/mL for 1 h	*OGG1, Top3a, Rad18*	RT-PCR	Significant down-regulation	Gadhia et al. (2012) [9]
CL3 human lung cancer cells	Lead acetate	10–100 µM for 30 min, and 24 h	*APE1*	Western blot	Significant increase in APE1 protein level in a dose-dependent manner	Wang et al. (2013) [34]
Stem cells from dental origin	Lead nitrate	160 µM for 24 h	*ERCC3, XRCC4, RAD51*	RT-PCR	No significant change	Abdullah et al. (2014) [33]
Lymphoblastoid TK6 cells	Lead acetate	120 µM for6–24 h	*XRCC1, hOGG-1, BRCA1, XPD*	Western blot	Significant decreases in protein levels of XRCC1 at 12 h; hOGG-1 at 6, 12, and 24 h; BRCA1 at 12 and 24 h; and XPD at 6 and 12 h	Liu et al. (2018) [31]
Roots of *A. cepa var. agrogarum*	Lead nitrate	5.0 and 15.0 µM for 12 h	*POLD1*	RT-PCR and MS	Significant down-regulation	Lyu et al. (2020) [32]

**RT-PCR**: reverse transcription-polymerase chain reaction; **MS**: mass spectrometry; **OGG1**: 8-oxoguanine DNA glycosylase 1; **Top3a**: DNA topoisomerase 3-alpha; **Rad18**: E3 ubiquitin-protein ligase RAD18; **APE1**: AP endonuclease; **ERCC3**: Excision Repair 3; **XRCC4**: X-ray repair cross-complementing protein 4; **RAD51**: RAD51 Recombinase; **XRCC1**: X-ray repair cross-complementing protein 1; **BRCA1**; Breast Cancer gene 1; **XPD**: xeroderma pigmentosum group D; and **POLD1**: DNA Polymerase Delta 1.

### 3.2. Epidemiological Studies

To date, there are only three studies that have evaluated the expressions of DNA repair genes in human populations exposed to Pb. Two of them were performed in occupationally exposed workers, and one study was performed on residents with a history of long-term exposure to the environmental metals as shown in Table 2.

A previous cohort study showed a significant decrease of OGG1-2a in Pb-exposure in workers from a construction site when compared with age-matched controls [35]. Moreover, Singh et al. (2020) reported significantly higher blood lead levels (BLL) and significant down-regulation of OGG1, XRCC1, and XPD in Pb-exposed workers as compared with the unexposed group [36]. However, a negative result for the effect of Pb on the DNA repair gene expression was documented. Bakheet et al. (2013) demonstrated that long-term exposure to Pb did not show any significant changes in the mRNA expression of OGG1 and APE1 in the Pb-exposed residents of Mahd Adh Dhahab, Saudi Arabia [37].

**Table 2:** Epidemiological studies on the effects of Pb on the expression of DNA repair-related genes.

Subject	N	Blood Pb Level (µg/dL) (Mean ± SEM)	DNA Repair Gene	Method	Result	Reference
Workers of construction area origin	100 exposed 100 controls	-	*OGG1-2a*	RT-PCR	Significant down-regulation	Akram et al. (2019) [35]
Welding, handicraft, and paint workers	100 exposed 100 controls	7.88 ± 1.27 1.27 ± 0.11	*OGG1, XRCC1, XPD*	RT-PCR	Significant down-regulation	Singh et al. (2020) [36]
Exposed residents	40 exposed 20 controls	2.10 ± 0.25 1.12 ± 0.06	*OGG1, APE1*	RT-PCR	No significant change	Bakheet et al. (2013) [37]

**RT-PCR**: reverse transcription-polymerase chain reaction; **OGG1**: 8-oxoguanine DNA glycosylase 1; **APE1**: AP endonuclease; **XRCC1**: X-ray repair cross-complementing protein 1; **XPD**: xeroderma pigmentosum group D.

## 4. Discussion

Pb exposure potentially causes genotoxic effects, such as DNA breaks, chromosome aberrations, mutations, and the inhibition of DNA processing and repairs [38]. Several studies demonstrated the end-points of the genotoxic effect of Pb in different in vitro, in vivo, and epidemiological studies. They found that Pb exhibited positive responses in different biological and biochemical tests for DNA lesions, DNA mutations, and DNA oxidative damage [2]. Although the exact genotoxic properties and their molecular mechanisms are not fully elucidated, the previous studies suggested that Pb exerts its genotoxic action mostly through indirect mechanisms, such as the inhibition of DNA repair or DNA oxidative stress [2,6,16,17].

The DNA repair machinery plays a crucial role in protecting cells from damage generated by exposure to carcinogens and cytotoxic agents, as well as heavy metals. The DNA repair mechanisms include base excision repair (BER), nucleotide excision repair (NER), mismatch repair (MMR), homologous recombination (HR), nonhomologous end joining (NHEJ), translesion synthesis, and DNA interstrand crosslink repair [1]. The different DNA repair systems recognize specific types of isolated damages; however, they may interact in the repair of complex damages, for example, clustered DNA damage.

BER involves repairing isolated damages and non-bulky lesions, such as single-strand breaks, single base damage, and oxidative lesions. MMR repairs replication errors and other base mismatches. However, NER is responsible for complex damage and removing bulky lesions. HR and NHEJ correct double-strand breaks. Translesion synthesis bypasses intrastrand crosslinks and bulky lesions, and DNA inter-strand crosslink repair removes interstrand crosslinks [39,40]. In the DNA repair process, cells are equipped with intricate and sophisticated systems—DNA repair, damage tolerance, cell cycle checkpoints, and cell death pathways—which collectively function to reduce the deleterious consequences of DNA damage.

The studies reviewed in this article evidenced the impact of Pb on the expression of DNA repair genes in in vitro and epidemiological studies. Most studies reviewed in this article demonstrated a decrease in the expression of DNA repair genes in the BER, NER, and double-strand break repair pathways. According to the studies reviewed, DNA repair genes OGG1, APE1, and XRCC1, known to play roles in the BER pathway, were reported to be down-regulated by Pb toxicity. BER is responsible for correcting the oxidized base damage [39]. Thus, these findings support the previous studies demonstrating Pb-induced oxidative DNA damage, a common DNA damage lesion induced by Pb toxicity. In the NER pathway, DNA repair genes XPD and POLD1 were found to be down-regulated by Pb. NER has a key role in removing bulky DNA lesions [40], which can occur under heavy metal toxicity as a result of elevated ROS levels under oxidative stress [41]. According to previous studies, DNA double-strand break damage was detected under Pb toxicity [32,42]. Interestingly, the DNA repair gene, BRCA1, known to play an important role in the homologous recombinant double-strand break repair pathway, was reported to be down-regulated by Pb. Although there was an altered expression of DNA repair genes, Abdullah et al. (2014) and Bakheet et al. (2013) reported no significant changes in the expression of DNA repair genes. However, Abdullah et al. suggested factors that act as confounding factors and need to be addressed so as to avoid misinterpretation. For example, prolonged duration times of exposure should be considered to cover sub-chronic and chronic effects in the in vitro models [34]. According to all of the epidemiological studies reviewed in this article, there was significant down-regulation of the DNA repair gene expression and high blood Pb levels in exposed groups as compared to control groups, except for in the work of Bakheet et al., who reported no significant changes in the expression of DNA repair genes in the exposed volunteers, which might be due to the normal blood Pb levels of the exposed group [36]. Therefore, it can be suggested that Pb accumulation levels may be associated with the expression level of DNA repair genes.

Overall, the studies reviewed in this article presented a significant down-regulation of DNA repair genes, which is consistent with previous studies demonstrating the association of the aberrant expression of DNA repair genes with cadmium and lead. For example, Zhou et al. reported decreased expressions of DNA repair genes XRCC1, ERCC1, and hOGG1 with the cadmium (Cd)-induced malignant transformation of human bronchial epithelial cells [43]. Moreover, XRCC1, hOGG1, and ERCC1 significantly declined in the liver, kidney, heart, and lung tissues of Cd-exposed rats as compared with the control group [44]. According to previous epidemiological studies, significant decreases in the expression of DNA repair genes hOGG1 and XRCC1 were observed in groups of cigarette and water pipe smokers with high BLL when compared to the control group [27]. The expression of OGG1 was significantly down-regulated in an occupational heavy metals-exposed group with significantly higher blood Pb and Cd levels as compared with the control group [44].

Although the molecular mechanisms of Pb toxicity related to the inhibition of DNA repair systems remain unclear, epigenetic mechanisms have been suggested as being involved in the abnormal regulation and expression of DNA-related repair genes under heavy metal toxicity. Epigenetics involves gene expression and regulation without DNA sequence changes [45]. Transcriptional regulation is administered through important epigenetic pathways, dictated primarily by DNA methylation, RNA regulation, and the post-translational modification (PTM) of histones [8]. Liu et al. reported that the expression of DNA repair genes was inhibited via enhancing their promoter methylation in TK6 cells after exposure to Pb [31]. Moreover, several previous studies demonstrated an interaction between heavy metals and the aberrant expression of DNA repair genes via epigenetic mechanisms, such as aberrant DNA methylation, modified histone modification, altered expression profiles of microRNAs (miRNAs), and long non-coding RNAs (lncRNAs) [9,10,11,31,45]. Based on the concept that epigenetics has potential for better understanding the molecular mechanisms whereby environmental metal exposure leads to heritable epigenetic marks, correlating with gene expression patterns, future studies should be planned to investigate the epigenetic mechanisms that may play an important role in the alteration of DNA repair gene expression under Pb toxicity.

Future studies should be planned to validate these findings in different test systems, and other DNA repair genes need to be explored. Furthermore, the experimental variables acting as confounding factors and influencing the variability in these studies, such as dose, duration time, route of Pb exposure, multiple exposures to other genotoxic agents, smoking habits, lifestyles, or types of cell lines, need to be taken into consideration. Finally, additional studies are needed to obtain a complete picture of the genotoxic properties of Pb and the underlying mechanisms that may have a crucial role in carcinogenesis.

## 5. Conclusions

Although evidence of the effect of Pb on DNA repair systems exists, research findings still conflict with each other. Future studies should be performed to evaluate these results in different test systems, and several DNA repair genes need to be explored to support the hypothesis that the genotoxic effects of Pb could be due to indirect mechanisms, such as the inhibition of DNA repair processes. Moreover, epigenetic mechanisms may have a crucial role in the alteration of DNA repair gene expression under Pb toxicity. The role of Pb toxicity in the impairment of DNA repair should be considered as a high risk of cancer development.
